# MET-mediated phosphorylation of YANK2 at Y282 inhibits NEDD4L-dependent SUMOylation and degradation, promoting chemoresistance in glioblastoma

**DOI:** 10.1186/s43556-026-00496-3

**Published:** 2026-06-24

**Authors:** Yue Shi, Yue Cheng, Wensheng Li, Annie Zhu, Juanjuan Xiao, Wei Wang, Liu Tang, Shuang Zhao, Mee-Hyun Lee, Olesya S. Malyarenko, Qiuhong Duan

**Affiliations:** 1 Department of Biochemistry and Molecular Biology, School of Basic Medicine, Tongji Medical College, Huazhong University of Science and Technology, Wuhan, Hubei 430030 China; 2 Translational Medicine Center, Huaihe Hospital of Henan University, Henan University, Kaifeng, Henan China; 3 Department of Clinical Laboratory, Zhengzhou Eighth People’s Hospital, Zhengzhou, Henan China; 4 Department of Blood Transfusion, Wuhan Fourth Hospital, Wuhan, 430000 Hubei China; 5 College of Korean Medicine, Dongshin University, Naju, 58245 South Korea; 6 Phillips Exeter Academy, PEA 2211, 20 Main Street, Exeter, NH03833 USA

**Keywords:** MET, YANK2, Phosphorylation, SUMOylation, Chemosensitization

## Abstract

Glioblastoma (GBM) is the most common and aggressive primary malignant brain tumor in adults, characterized by rapid progression and exceptionally poor prognosis. Identifying novel molecular drivers and therapeutic targets is urgently needed. This study reports a previously unrecognized MET–YANK2 signaling axis that drives glioma progression. Analysis of glioma patient samples reveals that high co‑expression of MET and YANK2 is positively correlated and significantly associated with poor survival outcomes. Mechanistically, MET directly phosphorylates YANK2 at tyrosine 282 (Y282), a conserved residue critical for maintaining YANK2 protein stability. This phosphorylation event prevents SUMOylation mediated proteasomal degradation of YANK2, thereby enhancing its oncogenic function. Functional assays demonstrate that YANK2 phosphorylation promotes GBM cell proliferation and tumor growth both in vitro and in vivo. Conversely, loss of this phosphorylation or enhanced SUMOylation at lysine residues K8 and K148 markedly suppresses YANK2 oncogenic activity. Through structure based screening, rutin, a natural flavonoid compound, is identified as a potent direct binder of YANK2. Rutin treatment effectively inhibits YANK2 kinase activity, reduces downstream p70S6K phosphorylation, and selectively suppresses proliferation of YANK2 high GBM cells. Importantly, rutin exhibits synergistic effects with temozolomide (TMZ), significantly inhibiting tumor growth and prolonging survival in YANK2 overexpressing orthotopic glioma models. Collectively, these findings establish YANK2 as a novel prognostic biomarker and a promising therapeutic target, and highlight rutin as a potential chemosensitizer for biomarker driven combination therapy in glioma.

**Supplementary Information:**

The online version contains supplementary material available at 10.1186/s43556-026-00496-3.

## Introduction

Glioblastoma (GBM) is the most common and aggressive malignant primary brain tumor in adults, exhibiting high invasiveness, pronounced heterogeneity, treatment resistance, and resistance to conventional therapies [1]. Current standard treatments include maximal safe surgical resection [2], radiotherapy [3], and TMZ chemotherapy [4]. However, patient outcomes remain poor, with a median survival of only 12–15 months and a five-year survival rate of < 6% [5]. The clinical management of GBM is complicated by multiple factors including intrinsic resistance, restrictive properties of the blood–brain barrier, frequent tumor recurrence, and significant treatment-related toxicities [6]. These challenges underscore the urgent need to better understand the molecular pathogenesis of GBM and develop novel therapeutic strategies to improve patient prognosis.

Recent genomic studies have identified extensive genetic and epigenetic alterations in GBM, including characteristic molecular changes such as loss of PTEN [7], TP53 mutations [8], receptor tyrosine kinase (RTK) amplification [9], CDKN2A/CDKN2B inactivation [10], and IDH1/2 mutations [11]. Notably, approximately 4% of GBM cases exhibit clinically relevant cellular-mesenchymal epithelial transition factor (MET) gene alterations, including METex14 skipping mutations [12], PTPRZ1-MET (ZM) fusion genes [13], MET amplification, and dysregulated circMET expression [14]. These molecular aberrations result in constitutive MET pathway activation, which drives glioma progression through the PI3K/AKT/mTOR and STAT3 signaling cascades [15, 16], and contributes to immunosuppressive microenvironment formation [17]. Notably, phase II clinical trials have demonstrated that combinatorial therapy with onartuzumab (a MET inhibitor) and bevacizumab failed to significantly improve outcomes in patients with GBM [12, 17]. These findings suggest that monotherapeutic MET blockade may have limited efficacy in controlling glioma progression, underscoring the need for deeper investigation of MET regulatory networks and alternative therapeutic strategies.

The serine/threonine kinase STK32B (YANK2) is a recently characterized kinase that regulates cellular functions through substrate protein phosphorylation [18]. Emerging evidence demonstrates YANK2’s involvement in brain development and angiogenesis, and its dysregulation is linked to neurological disorders, including essential tremor [19]. Recent studies revealed elevated YANK2 expression in GBM tissues, which was correlated with poor patient prognosis [20]. Mechanistically, YANK2 activity is regulated by Fyn-mediated phosphorylation at the Y110 residue, which subsequently activates the p70S6K signaling pathway to drive tumor progression [20]. Nevertheless, the comprehensive molecular regulatory network governing YANK2 function—particularly its potential crosstalk with RTK signaling pathways such as MET—remains poorly understood.

It was therefore hypothesized that MET may directly regulate YANK2 through site-specific phosphorylation, thereby establishing a previously unrecognized signaling axis that promotes GBM progression. To test this hypothesis, in vitro kinase screening assays, molecular and cellular analyses of YANK2 phosphorylation and SUMOylation, and high-throughput compound screening combined with structure-based approaches were performed. The results demonstrate for the first time that MET directly phosphorylates YANK2 at tyrosine 282 (Y282), establishing a novel MET–YANK2 signaling axis. Mechanistic studies further reveal that MET-mediated phosphorylation regulates YANK2 SUMOylation, uncovering previously unknown crosstalk between these post-translational modifications. Through compound screening, rutin was identified as a potent direct binder and inhibitor of YANK2. Functionally, rutin treatment suppresses YANK2 kinase activity, reduces downstream p70S6K phosphorylation, selectively inhibits proliferation of YANK2-high GBM cells, and synergizes with temozolomide to suppress tumor growth and prolong survival in orthotopic glioma models. Collectively, these findings not only advance the understanding of GBM pathogenesis but also lay the foundation for biomarker-driven combination therapies targeting the MET–YANK2 axis.

## Results

### MET directly phosphorylates YANK2 to drive glioma progression

**Fig. 1:**
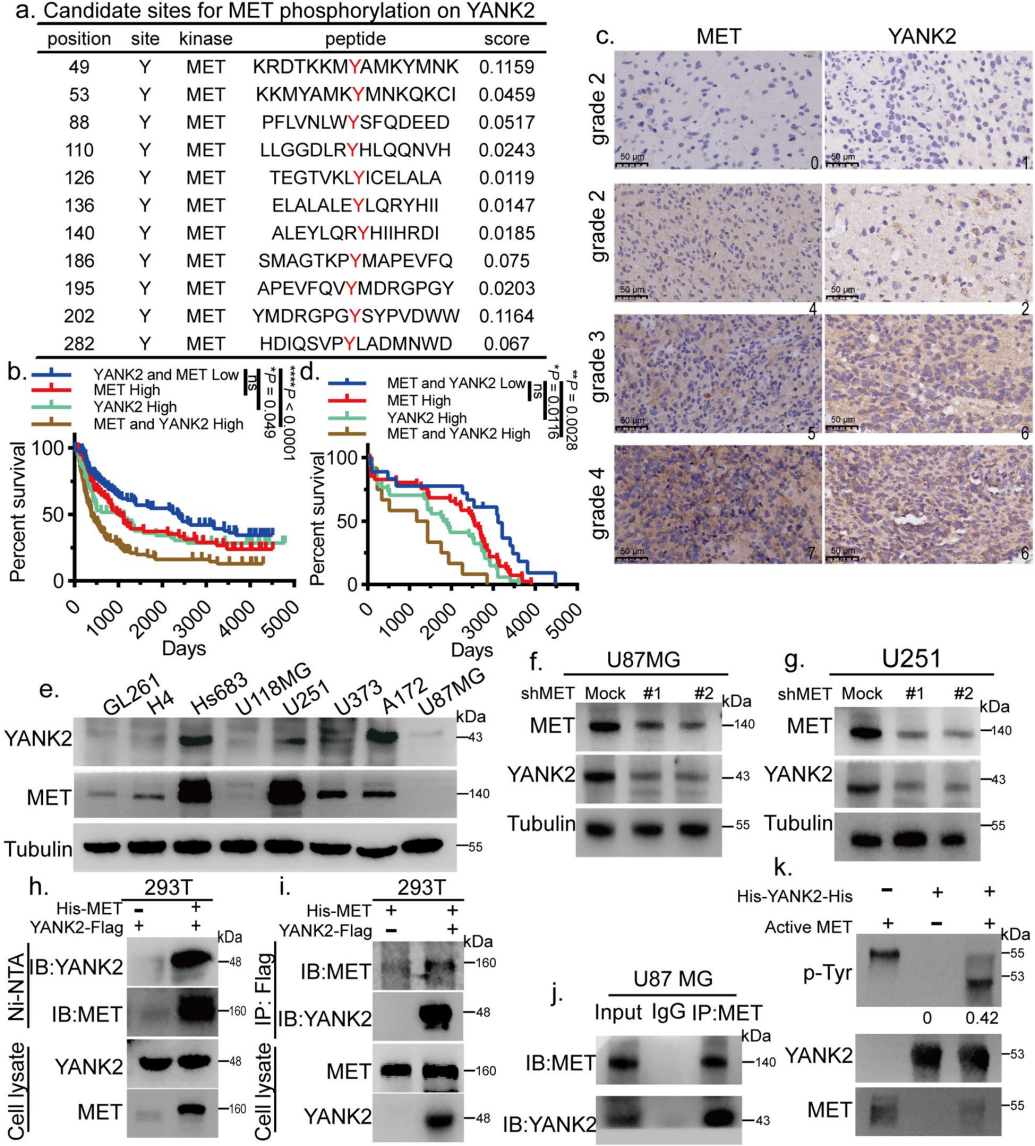
MET directly phosphorylates YANK2 to drive glioma progression. **a** Based on prediction by the GPS website, MET is a potential upstream kinase of YANK2. **b** Kaplan–Meier survival curves of GBM patients stratified by MET and YANK2 mRNA levels in the CGGA dataset (MET and YANK2 low, n = 93; YANK2 high, n = 67; MET high, n = 61; MET and YANK2 high, n = 23). **c** Representative immunohistochemistry (IHC) staining in 81 GBM patient samples. The numbers in the lower right corner of the images represent staining scores calculated based on positive staining area and staining intensity, Rabbit IgG was used as a negative control, showing no obvious positive staining. Scale bars: 25 μm. **d** Kaplan–Meier survival curves of GBM patients stratified by protein expression of MET and YANK2 (MET and YANK2 low, *n* = 20; YANK2 high, *n* = 26; MET high, *n* = 17; MET and YANK2 high, *n* = 18). **e** Western blot analysis showing MET and YANK2 expression in eight glioma cell lines. **f**-**g** Western blot analysis of MET and YANK2 protein levels in U87 MG and U251 shMET glioma cell lines. **h**, **i** Co-immunoprecipitation (co-IP) of MET-His and YANK2/His-YANK2-Flag in HEK293T cells using Ni–NTA or anti-Flag beads, followed by WB analysis. **j** Endogenous co-IP of YANK2 in whole lysates from U87MG cells using anti-YANK2 or IgG, followed by WB. **k** In vitro kinase assay showing phosphorylation of His-YANK2-His by recombinant active MET

Using the GPS web-based prediction tool (http://gps.biocuckoo.cn), we identified MET as a potential upstream kinase of YANK2 (Fig. 1a). To investigate the clinical association between MET and YANK2 expression in gliomas, we first performed an mRNA correlation analysis using the CGGA database. Pearson correlation analysis revealed a significant positive correlation between MET and YANK2 mRNA levels (Fig. S1c). Patients with high co-expression of MET and YANK2 exhibited significantly shorter overall survival compared to those with low co-expression (Fig. 1b; Fig. S1a–b). To validate these findings at the protein level, we performed immunohistochemical (IHC) staining on tumor tissues from 81 glioma patients. IHC scoring (based on staining intensity and percentage of positive cells) confirmed a positive correlation between MET and YANK2 protein expression (Fig. 1c; Fig. S1d). Consistently, patients with high protein levels of both markers had worse outcomes (Fig. 1d). Furthermore, multivariate Cox regression analysis using CGGA data showed that YANK2 expression level remained an independent prognostic factor after adjusting for age and IDH1 mutation status (Fig. S1e). Western blotting was then used to screen MET and YANK2 expression across eight glioma cell lines. Both proteins were highly expressed in Hs683, U87MG, and U251 cells, but weakly expressed in GL261 and U118MG cells (Fig. 1e). Together, these data suggest that MET and YANK2 expression are positively correlated at both mRNA and protein levels, and their high co‑expression may be associated with poor survival in glioma patients.

Having established the clinical correlation, we next sought to elucidate the regulatory relationship between MET and YANK2. To determine whether MET affects YANK2 protein expression, we generated MET‑silenced U251 and U87MG cells using short hairpin RNA (shRNA). MET knockdown led to a marked reduction in YANK2 protein levels (Fig. 1f–g). To test whether this regulation occurs at the transcriptional level, we measured YANK2 mRNA expression in MET‑knockdown cells by quantitative real‑time PCR (qRT-PCR). No significant change in YANK2 mRNA was observed (Fig. S1f), suggesting that MET regulates YANK2 post‑translationally. Next, to examine whether MET physically interacts with YANK2, we performed co‑immunoprecipitation (co‑IP) assays. Both exogenous (overexpressed tagged proteins) and endogenous (native proteins) co‑IP experiments demonstrated a direct interaction between MET and YANK2 (Fig. 1h–j). Finally, to test whether MET directly phosphorylates YANK2, we conducted an in vitro kinase assay using recombinant YANK2 protein purified from E. coli and active MET kinase. The result showed that MET directly phosphorylates YANK2 (Fig. 1k). Collectively, these findings suggest that MET acts as an upstream kinase of YANK2 and regulates YANK2 protein through direct phosphorylation, a post‑translational modification, rather than through transcriptional control.

### The phosphorylation of YANK2 by MET promotes GBM growth in vitro and in vivo

**Fig. 2:**
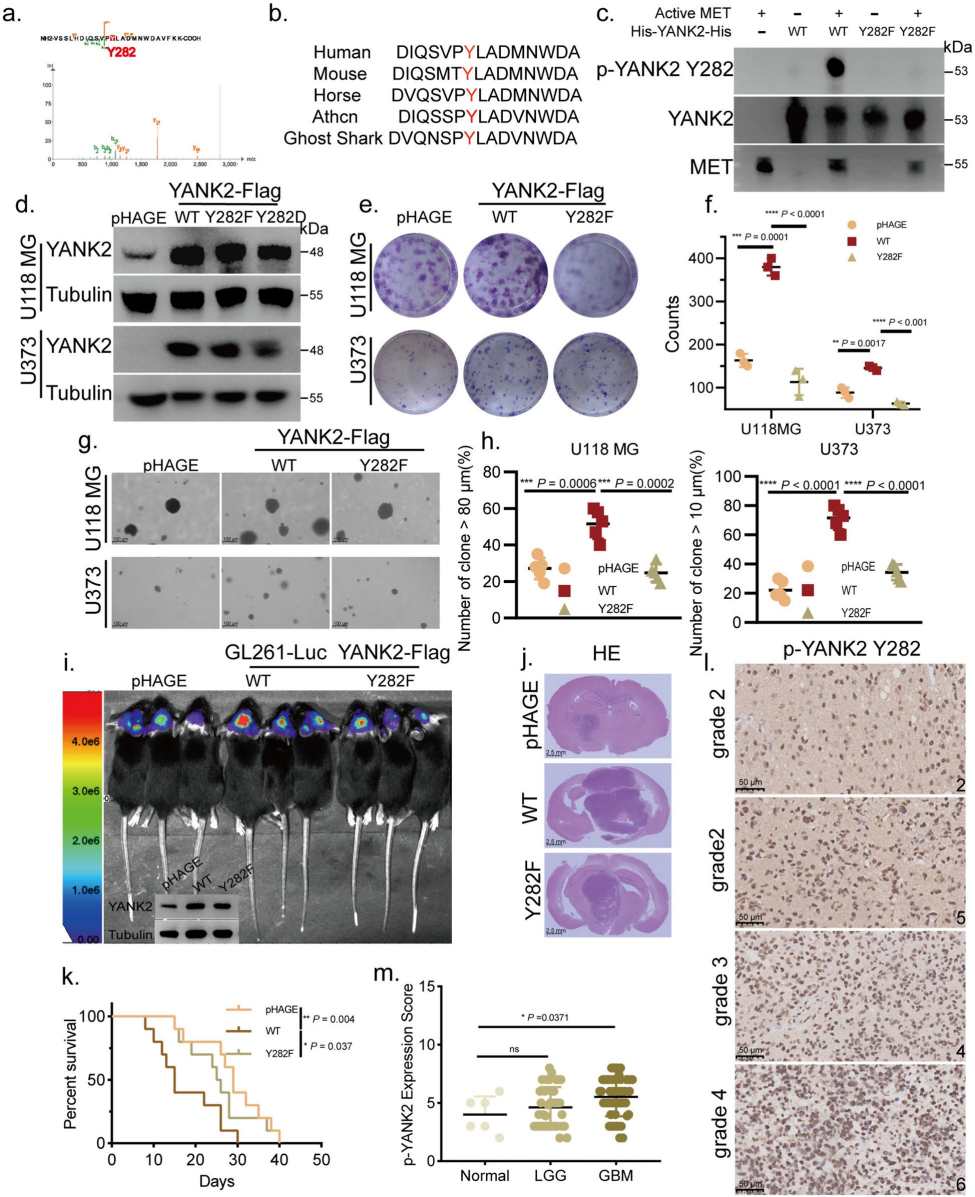
MET-mediated phosphorylation of YANK2 promotes GBM growth in vitro and in vivo. **a** Annotated LC–MS/MS spectrum of the phosphorpeptide containing the Y282 site of YANK2. **b** Conservation of the tyrosine residue Y282 across species. **c** Kinase assay confirming MET phosphorylates YANK2-WT but not YANK2-Y282F mutant, detected with a p-YANK2-Y282 antibody. **d** Validation of stable overexpression of YANK2-WT, Y282F or Y282D mutants by WB. **e**, **f** Colony formation assay in U118MG/U373 cells stably expressing YANK2-WT or Y282F-Flag, and the quantitative analysis of colony numbers. **g**, **h** Soft agar assay in U118MG/U373 cells stably expressing YANK2-WT or Y282F-Flag, and the quantitative analysis of colony numbers. scale bar: 100 μm. **i** Representative bioluminescent images of C57 BL/6 mice bearing orthotopic tumors from GL261-YANK2-WT or Y282F cells at day 14 (n = 8 per group). **j** Quantification of representative H&E-stained brain sections of tumor xenografts, scale bar: 2.5 mm. **k** Kaplan–Meier survival curves for orthotopic glioma-bearing mice (n = 8 per group). **l**, **m** Representative IHC images of p-YANK2 (Y282) expression in 81 glioma samples and 5 nomal samples, the numbers in the lower right corner of the images represent staining scores calculated based on positive staining area and staining intensity (**l**) and quantification of positive scoring (**m**), Rabbit IgG was used as a negative control, showing no obvious positive staining. (Nomal, n = 5; LGG, n = 23; MET high, n = 58; MET and YANK2 high, n = 18). scale bar: 25 μm

To identify the specific residue(s) on YANK2 phosphorylated by MET, we performed liquid chromatography‑tandem mass spectrometry (LC‑MS/MS) on YANK2 protein incubated with active MET kinase in vitro. Mass spectrometry identified tyrosine 282 (Y282) as a specific phosphorylation site targeted by MET (Fig. 2a). Sequence alignment revealed that Y282 is highly conserved across multiple species, suggesting its functional importance (Fig. 2b). To further test this finding, we conducted in vitro kinase assays using wild‑type (WT) YANK2 and a Y282F mutant (in which tyrosine is replaced by phenylalanine, abolishing phosphorylation at this site). The Y282F mutant exhibited significantly reduced phosphorylation compared to WT, confirming that MET directly phosphorylates YANK2 at Y282 (Fig. 2c). Consistently, levels of p‑YANK2 (Y282) were also reduced in MET‑knockdown (shMET) cell lines (Fig. S1g‑h). Having identified Y282 as the key MET phosphorylation site, we next investigated its functional role in glioma cell proliferation and tumor growth. We established stable human glioma cell lines expressing YANK2 WT, the non‑phosphorylatable mutant Y282F, or the phospho‑mimetic mutant Y282D (in which aspartate mimics constitutive phosphorylation) (Fig. 2d). Colony formation assays and soft agar showed that cells expressing Y282F exhibited significantly reduced proliferative capacity compared to WT‑expressing cells (Fig. 2e‑h). To further validate these findings in an independent model, we generated YANK2 (WT and Y282F) overexpressing cell lines in mouse GL261-Luc glioma cells. We then assessed the impact of Y282 phosphorylation on tumor growth in vivo using an orthotopic mouse model implanted with GL261-Luc cells. Mice bearing Y282F‑derived tumors showed significantly slower tumor growth and prolonged survival compared to those bearing WT‑expressing tumors (Fig. 2i-k). Finally, to assess clinical relevance, we performed immunohistochemical (IHC) staining for p‑YANK2 (Y282) in human glioma tissue samples. Higher levels of p‑YANK2 (Y282) were detected in high‑grade glioma tissues compared to low‑grade tissues (Fig. 2l‑m; *P* = 0.0371). Collectively, these results suggest that MET‑mediated phosphorylation of YANK2 at tyrosine 282 may promote glioma progression by enhancing cell proliferation and tumor growth.

### Phosphorylation of YANK2 stabilizes the protein by preventing SUMO-mediated proteasomal degradation

**Fig. 3:**
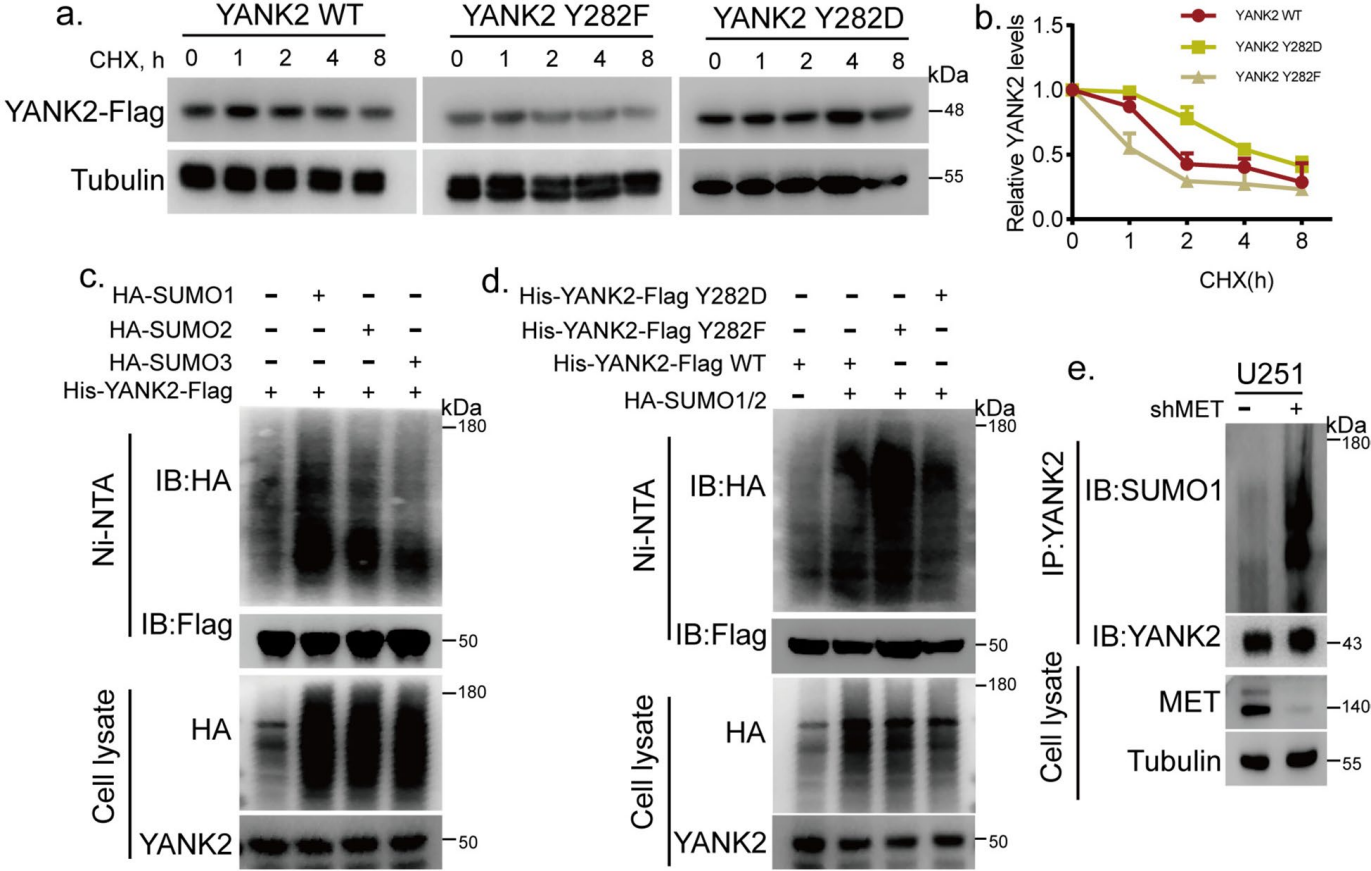
MET-mediated phosphorylation of YANK2 inhibits its SUMO-mediated proteasomal degradation. **a**–**b** CHX chase assay showing degradation kinetics of YANK2-WT versus Y282F mutant in U118MG stable cell lines; (b) shows quantification of band intensity. **c** WB analysis of SUMOylation of YANK2 in HEK293T cells transfected with SUMO1, SUMO2 and SUMO3 plasmids. **d** WB analysis of SUMOylation of YANK2(WT, Y282F, Y282D) in HEK293T cells transfected with SUMO1 and SUMO2 plasmids. **e** U251 mock and shMET cells were subjected to immunoprecipitation (IP) with anti-YANK2 antibody, followed by western blot (WB) analysis with anti-SUMO antibody to detect SUMOylation levels of YANK2

To determine whether MET-mediated phosphorylation at Y282 affects YANK2 protein stability, we performed cycloheximide (CHX) chase assays. CHX is a protein synthesis inhibitor used to measure the degradation rate of existing proteins. As shown in Fig. 3a and b, the half-life of the YANK2-Y282D phosphomimetic mutant was significantly longer than that of the Y282F non-phosphorylatable mutant, indicating that phosphorylation at Y282 enhances protein stability. To investigate the degradation pathway involved, we treated cells with Z-Leu-Leu-Leu-al **(**MG132), a proteasome inhibitor, which led to YANK2 accumulation, suggesting that the proteasome pathway mediates YANK2 degradation (Fig. S2a). Unexpectedly, co-immunoprecipitation assays demonstrated that YANK2 was not ubiquitinated but instead underwent SUMOylation (Fig. 3c and Fig. S2b). SUMOylation is a post-translational modification that covalently attaches small ubiquitin-like modifier (SUMO) proteins to target proteins, often regulating protein stability or localization [21]. The Y282F mutation increased YANK2 SUMOylation levels (Fig. 3d). Furthermore, in MET-knockdown cell lines, MET depletion resulted in elevated YANK2 SUMOylation (Fig. 3e). Collectively, these results indicate that phosphorylation at Y282 blocks SUMO modification, thereby preventing proteasome-mediated degradation of YANK2.

### NEDD4L may act as a potential E3 ligase promoting YANK2 SUMOylation and degradation

**Fig. 4:**
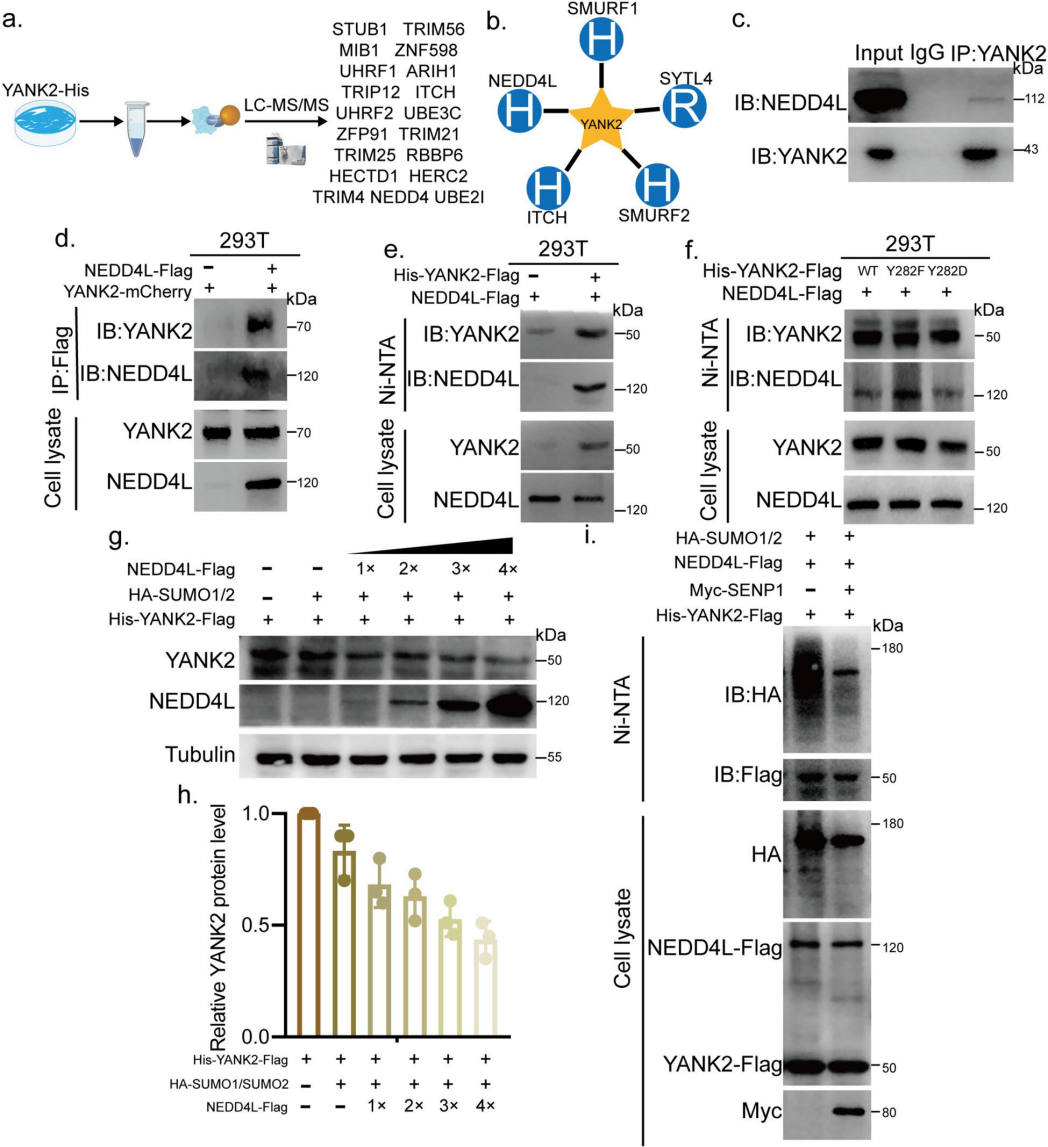
NEDD4L is a potential E3 ligase responsible for YANK2 SUMOylation. **a** Schematic of YANK2 interactome screening by IP-MS/MS with E3 ligases identified shown. **b** Network prediction of potential YANK2 E3 ubiquitin ligases. **c** Co-IP of U87MG whole lysates with anti-YANK2 or IgG, followed by WB. **d**, **e** Co-precipitation with anti-Flag or Ni–NTA pull-down of His-YANK2-Flag/YANK2-mCherry and NEDD4L-Flag co-expressed in HEK293T cells, followed by WB. **f** Co-precipitation with Ni–NTA pull-down of His-YANK2 WT/Y282F/Y282D-Flag and NEDD4L-Flag co-expressed in HEK293T cells, followed by WB. **g**, **h** WB analysis showing dose-dependent effects of NEDD4L-Flag on YANK2 levels; quantification normalized to control. **i** WB analysis of SUMOylation of YANK2 in HEK293T cells transfected with SUMO1/2, NEDD4L and SENP1 plasmids

To identify the E3 ligases responsible for YANK2 SUMOylation, we first enriched His-YANK2-interacting proteins using Ni–NTA resin—a nickel-charged affinity matrix that binds histidine-tagged proteins—and analyzed them by mass spectrometry. IP-LC–MS analysis identified 19 candidate E3 ligases interacting with YANK2 (Fig. 4a). E3 ligases are enzymes that facilitate the transfer of ubiquitin or ubiquitin-like molecules such as SUMO to target proteins. Bioinformatic screening using UbiBrowser, a database for predicting ubiquitination-related E3 ligases, predicted five potential E3 ligases: NEDD4L, ITCH, SMURF1, SMURF2, and SYTL4 (Fig. 4b). However, co-immunoprecipitation (co-IP) assays with a YANK2 antibody detected binding between YANK2 and NEDD4L but not ITCH (Fig. 4c). Given the homology between NEDD4 and NEDD4L, we focused on NEDD4L. Co-IP assays further confirmed a direct interaction between YANK2 and NEDD4L (Fig. 4d–e). Comparing wild-type (WT), Y282F, and Y282D mutants, we found that the Y282F mutant exhibited the strongest binding to NEDD4L (Fig. 4f). In the presence of SUMO1/2, increasing expression of NEDD4L decreased YANK2 protein levels in a dose-dependent manner (Fig. 4g–h), supporting that NEDD4L promotes YANK2 degradation via SUMOylation. To test whether NEDD4L directly mediates YANK2 SUMOylation, we first confirmed that YANK2 interacts with UBC9, the SUMO E2-conjugating enzyme (Fig. S2d). Moreover, in the presence of co-transfected NEDD4L and SUMO, the deSUMOylating enzyme SENP1 significantly suppressed YANK2 SUMOylation (Fig. 4i). Collectively, these results suggest that NEDD4L is a key E3 ligase mediating YANK2 SUMOylation.

### SUMOylation at K8 and K148 sites of YANK2 inhibits glioblastoma progression

**Fig. 5:**
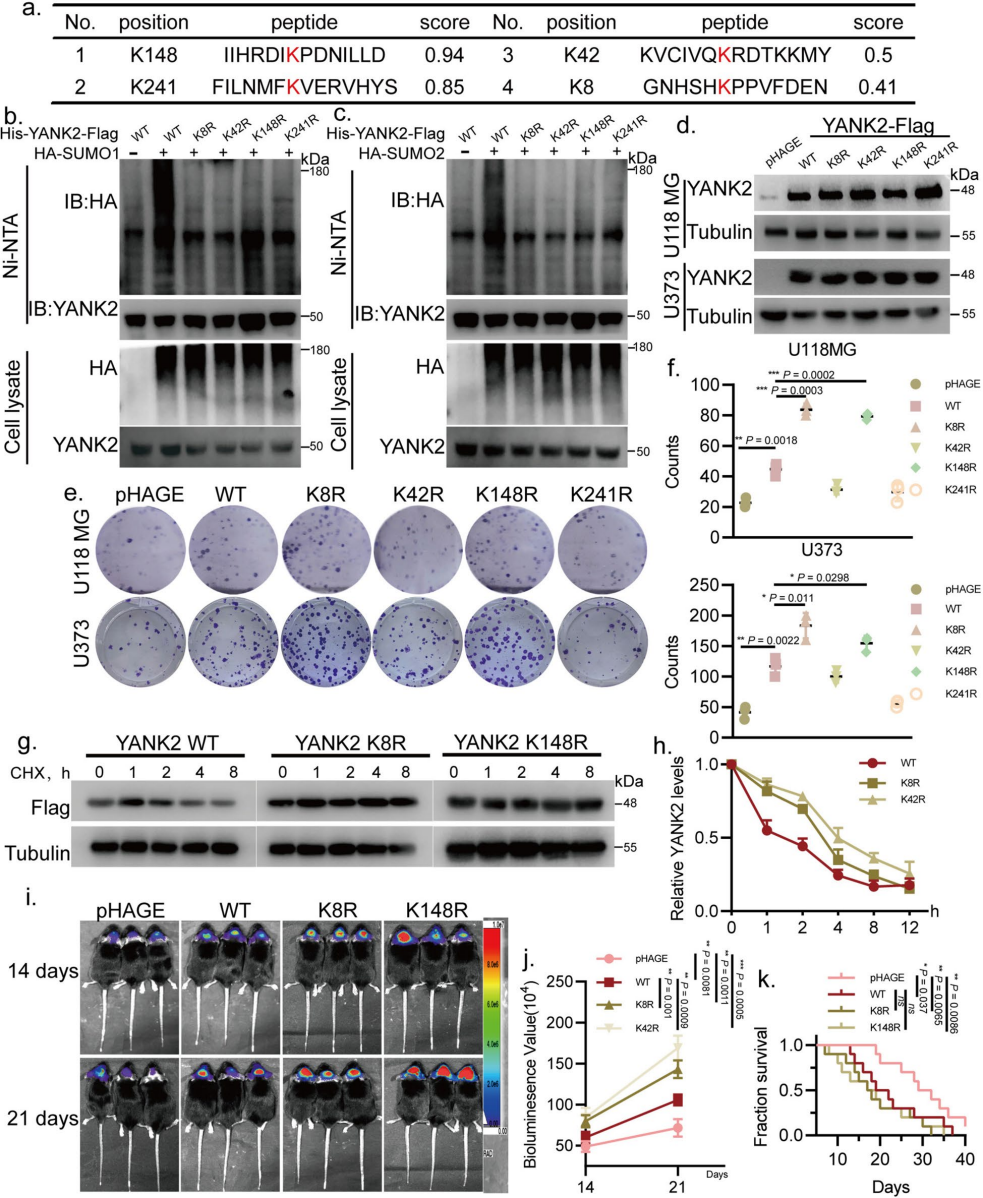
SUMOylation at K8 and K148 sites of YANK2 inhibits glioblastoma progression. **a** Predicted YANK2 SUMOylation sites. **b**, **c** WB analysis of SUMO levels in HEK293T cells expressing WT or K mutant YANK2 plasmids. **d** Validation of stable overexpression of YANK2-WT, K8R, K42R, K148R or K241R mutants in U118 MG and U373 by WB. **e**, **f** Colony formation assay of WT and K mutant cell lines (14 and 21 days) the quantitative analysis of colony number. **g**, **h** CHX chase assay showing degradation of YANK2-WT, K8R, or K148R mutants in U118MG stable lines and the quantitative analysis of YANK2 protein remaining at each time point normalized to the 0 h time point. **i** Bioluminescent images of C57BL/6 mice with tumors derived from GL261-YANK2-WT or K mutants at day 14 (n = 8 per group). **j** Quantitative analysis of tumor fluorescence intensity in orthotopic glioma-bearing mice (n = 6–8 per group). **k** Kaplan–Meier survival curves of the same mice (n = 8 per group)

To map the specific lysine residues on YANK2 that undergo SUMOylation, we performed SUMOplot analysis, a bioinformatic tool that predicts potential SUMOylation sites based on consensus motifs. This analysis identified four lysine residues as candidate SUMOylation sites (Fig. 5a). SUMOylation is the covalent attachment of small ubiquitin-like modifier (SUMO) proteins to lysine residues, which can regulate protein stability, localization, or function. Using site-directed mutagenesis to replace each lysine with arginine (K-to-R mutation, which prevents SUMO conjugation) and Ni–NTA pull-down assays, we confirmed that all four sites were indeed SUMOylated (Fig. 5b–c). To investigate the functional consequences, we established stable U118 MG and U373 glioma cell lines expressing wild-type (WT) or individual K → R mutants (Fig. 5d). Colony formation assays revealed that the K8R and K148R mutants exhibited significantly enhanced proliferative capacity compared with WT (Fig. 5e–f). Cycloheximide (CHX) chase assays—which measure protein degradation rates by blocking new protein synthesis—showed that the half-life (t_1/2_) of both K8R and K148R mutants was prolonged, consistent with reduced proteasomal degradation (Fig. 5g–h). In vivo, GL261 glioma cells expressing the K8R or K148R mutants formed larger tumors and led to worse survival outcomes in C57BL/6 mice compared with WT controls (Fig. 5i–k and Fig.S2e). Collectively, these findings suggest that SUMOylation at lysine residues K8 and K148 may suppress YANK2 protein stability and inhibit glioma growth.

### Identification of rutin as a direct YANK2-binding inhibitor and its functional consequences

**Fig. 6:**
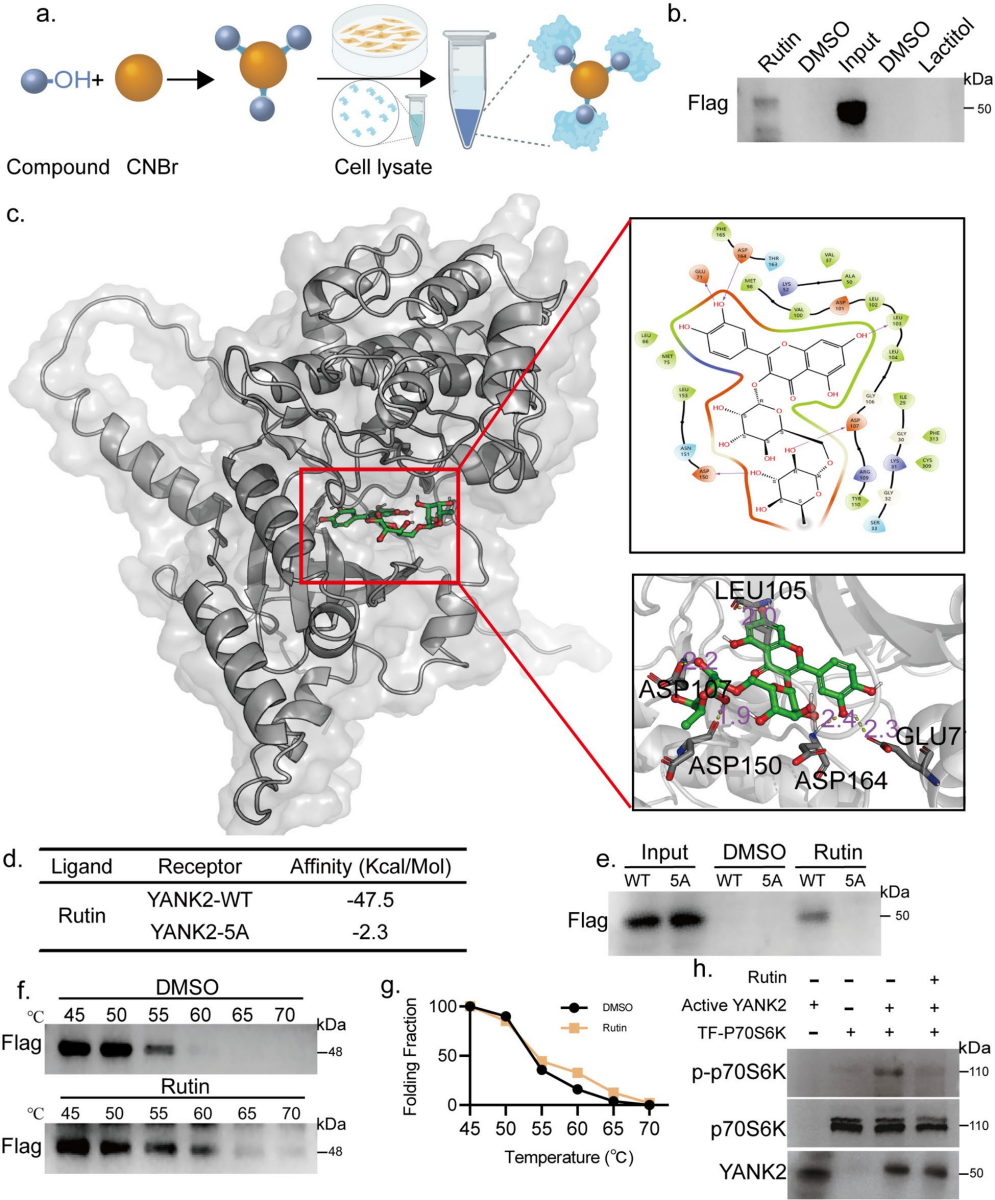
Computational screening identifies Rutin as a potential YANK2-targeting therapeutic. **a**, **b** Small molecule binding: schematic of co-incubation of Rutin-Sepharose 4B beads with U118MG-YANK2 lysate (**a**); WB detection of YANK2 in pull-down samples (**b**). **c** Predicted molecular docking structure of YANK2 (gray) with Rutin (colored); 2D interaction diagram of key residues. **d** Binding affinity comparison between Rutin and YANK2-WT versus YANK2-5A mutant (E71A, L103S, D105A, D150A, D164A). **e** Rutin pull-down assay confirming loss of binding in the 5 A mutant. **f**-**g** Recombinant YANK2 protein was incubated with DMSO (control) or rutin at the indicated temperatures, followed by western blot analysis to detect YANK2 protein levels and the quantitative analysis of relative YANK2 protein intensity. **h** In vitro kinase assays showing Rutin inhibits YANK2-mediated phosphorylation of p70S6K. The three-dimensional structure of YANK2 was obtained by AlphaFold prediction under the entry ID AF-Q9NY57-F1-v6

To identify potential small-molecule inhibitors targeting YANK2, we performed a structure-based virtual screening of 3,158 FDA-approved drugs. Virtual screening is a computational method used to predict and rank the binding affinity of small molecules to a target protein structure. This screening identified rutin hydrate and lactitol as the top candidates for YANK2 binding (Fig. S3a–b). To validate this interaction experimentally, we conducted pull-down assays, which confirmed direct binding of rutin to YANK2 (Fig. 6a–b). Next, to characterize the molecular determinants of this interaction, molecular dynamics simulations using AutoDock and AMBER force fields—computational tools that simulate atomic motions and calculate binding energies—identified five critical YANK2 residues mediating rutin binding: E71, L103, D105, D150, and D164 (Fig. 6c). We then engineered a quintuple YANK2 mutant (YANK2-5A: E71A, L103S, D105A, D150A, and D164A) through charge-altering substitutions that preserve structural integrity but disrupt the predicted binding interface. Binding free energy calculations revealed a significant reduction in affinity for YANK2-5A compared to wild-type YANK2 (ΔΔG =  − 2.3 kcal/mol, Fig. 6d). This prediction was biochemically validated using pull-down assays, which demonstrated that the mutant protein completely lost its rutin-binding capability (Fig. 6e). Furthermore, thermal shift stability assays—which measure changes in protein melting temperature upon ligand binding—showed that rutin elevated the melting temperature of YANK2 to above 60 °C, indicating that rutin binding significantly enhances YANK2 thermal stability (Fig. 6f-g). Collectively, these results suggest that rutin is a small-molecule compound that directly binds to YANK2.

To determine whether rutin inhibits glioma cell proliferation through suppressing YANK2 kinase activity, we first examined the effect of rutin on YANK2 enzymatic function using an in vitro kinase assay. This assay measures the ability of YANK2 to phosphorylate its substrate p70S6K. Rutin inhibited YANK2-mediated phosphorylation of p70S6K (Fig. 6h). Additionally, we verified through in vitro kinase assays that rutin does not affect the kinase activity of MET (Fig. S4h). MTT and colony formation assays showed that rutin significantly reduced proliferation of YANK2-high cells (U251 and U87MG) but had minimal effects on YANK2-low U118MG cells (Fig. S4a–c). Soft agar assays, which assess anchorage-independent growth, further validated the anti-proliferative effects of rutin in YANK2-high cells (Fig. S4d). Furthermore, wound healing assay results indicated that rutin did not affect the invasion of glioma cells(Fig. S4e). We then treated YANK2-high U251 and U87MG cells with rutin and the MET agonist HGF. Western blot analysis confirmed that rutin suppressed HGF-stimulated p-p70S6K levels in both cell lines (Fig. S4f). In U251 cells, rutin reduced p-p70S6K expression in a dose-dependent manner; however, after knocking down YANK2 expression, the effect of rutin on p-p70S6K was diminished (Fig. S4g). Taken together, these results suggest that YANK2 is a key protein through which rutin exerts its anti-proliferative effects in glioma cells.

### Combination of rutin and TMZ synergistically inhibits glioma progression in YANK2-high cells

**Fig. 7:**
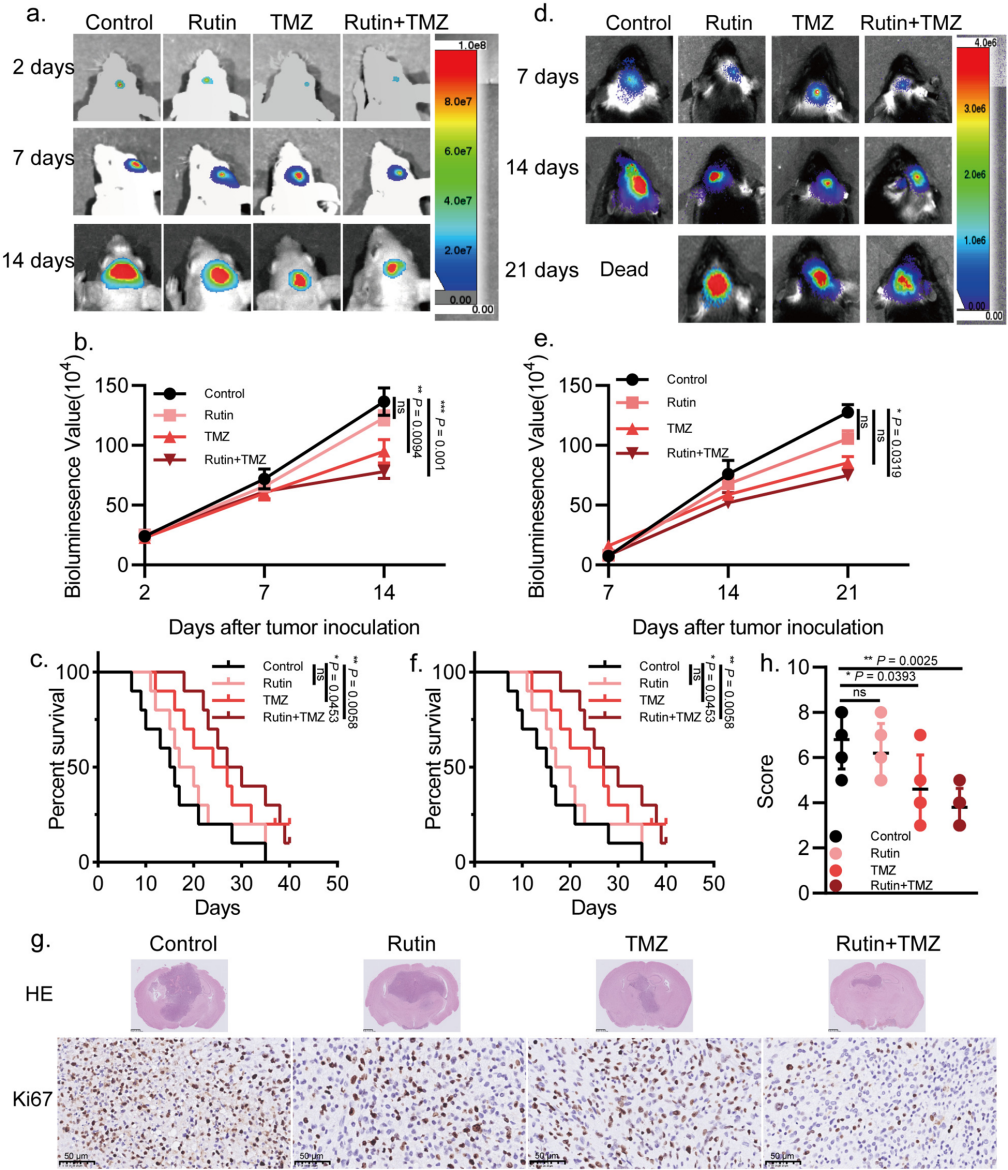
Combination treatment with Rutin and TMZ synergistically inhibits proliferation of YANK2-high glioma cells in vivo. **a**-**b** Representative in vivo bioluminescence images of intracranial GWHO4-Luc tumors treated in nude mice, Rutin (20 mg/kg), TMZ (45 mg/kg), or Rutin plus TMZ (n = 7 per group); quantification of relative tumor signal and survival curves, Representative images depict the same mouse per group. **c** Kaplan–Meier survival curves of the nude mice. **d**–**e** Representative in vivo bioluminescence images of intracranial U87MG-Luc tumors treated with vehicle in C57/BL mice, Rutin (20 mg/kg), TMZ (45 mg/kg), or Rutin plus TMZ (n = 8 per group); quantification of relative tumor signal and survival curves, Representative images depict the same mouse per group. **f** Kaplan–Meier survival curves of the C57/BL mice (n = 8 per group). **g**, **h** Representative H&E and Ki67 staining images of mouse brain sections and quantification of Ki67-positive cells, The numbers in the lower right corner of the images represent staining scores calculated based on positive staining area and staining intensity, Rabbit IgG was used as a negative control, showing no obvious positive staining. Scale bar: 3 mm, 25 μm

Combination treatment with rutin and TMZ significantly suppressed colony growth of YANK2-high U87MG and GWH04 cells (Fig. S4i), but had no significant effect on YANK2-low U118MG cells (Fig. S5a-b and Fig. S5e-h). These findings were further validated in GL261-YANK2 overexpression cell lines and U87MG shYANK2 cells (Fig. S5c-d and Fig. S5i-j). For in vivo validation, we first established a PDX model in nude mice using YANK2-high GWH04 cells to evaluate the therapeutic effect of rutin combined with TMZ. The results showed that rutin monotherapy failed to provide survival benefit to the mice, whereas combination treatment with rutin and TMZ significantly prolonged mouse survival compared with TMZ monotherapy (Fig. 7a-c). Subsequently, we established an orthotopic glioma model in C57BL/6 mice by intracranial injection of GL261-YANK2 cells (n = 8 per group). Longitudinal fluorescence imaging revealed a significant reduction in tumor fluorescence intensity in the combination therapy group (Fig. 7d-e). Survival analysis showed that TMZ monotherapy extended the median survival from 17 days (control group) to 24 days, while combination therapy further prolonged median survival to 28 days (*P* < 0.01 vs. TMZ alone) (Fig. 7f). Histopathological examination and Ki67 staining confirmed that the TMZ-rutin combination significantly inhibited tumor proliferation compared with monotherapy (Fig. 7g-h). Collectively, these results suggest that YANK2 serves as a predictive biomarker for rutin-mediated chemosensitization and provide a preliminary rationale for YANK2-targeted combination therapy for gliomas.

## Discussion

Although aberrant activation of the MET signaling pathway in GBM is well documented, therapeutic interventions targeting this pathway have yielded limited clinical success [22, 23]. Our study systematically investigated MET downstream effectors and made a novel discovery of YANK2 as a phosphorylation substrate, offering a potential breakthrough for overcoming the current treatment limitations. Importantly, YANK2 exhibits a distinctive expression pattern compared to established MET downstream molecules, such as GRB2 [24] and STAT3 [25, 26], showing minimal expression in normal tissues but marked upregulation in high-grade gliomas, which underscores its therapeutic potential. Our findings revealed that YANK2 phosphorylation at the Y282 site not only correlates with tumor aggressiveness, but also significantly promotes glioma cell proliferation, establishing this molecule as a key player in GBM progression.

A pivotal contribution of this study lies in deciphering the intricate post-translational modification (PTM) network governing YANK2 [27, 28]. Our findings demonstrate that MET-mediated phosphorylation at Y282 critically modulates YANK2 function by regulating its protein stability, offering novel mechanistic insights into how phosphorylation events drive tumor progression. Notably, we made the groundbreaking discovery that YANK2 undergoes SUMOylation rather than ubiquitination, with NEDD4L rather than classical PIAS family E3 ligases, which serve as mediating enzymes [29, 30]. The identification of K8 and K148 as the primary SUMOylation sites not only advances our understanding of the biological functions of NEDD4L but also provides fundamental new knowledge about SUMO modification mechanisms [31]. Notably, SUMOylation can serve as a degradation signal to promote ubiquitination and subsequent proteasomal degradation, and our findings provide a new foundation for further exploring the PTM regulatory network of YANK2. Mechanistically, SUMOylation-mediated proteasomal degradation often occurs via the SUMO-targeted ubiquitin ligase (STUbL) pathway, in which SUMO-interacting motifs (SIMs) enable specific recognition of SUMO-modified substrates by STUbL enzymes, followed by ubiquitination and degradation [32, 33]. Interestingly, a similar STUbL-like pathway has been reported in zebrafish, where NEDD4 (a homolog of NEDD4L) collaborates with the SUMO E2 enzyme UBC9 to mediate the degradation of DeltaNp63α [34]. Nevertheless, our data demonstrate that NEDD4L is an essential component of the SUMO-ubiquitin network in the context of YANK2 regulation. Based on these findings, we propose a working model: SUMOylation on YANK2 may serve as a “recognition signal” for an as-yet-unidentified SIM-containing “reader” protein. This model requires further experimental validation. Furthermore, immunohistochemistry analysis revealed nuclear accumulation of phosphorylated YANK2. Considering the role of SUMOylation in regulating subcellular localization, our data supports a model in which YANK2 may engage in signal transduction through modification-dependent nucleocytoplasmic trafficking [35, 36].

Furthermore, our findings demonstrated that YANK2 is phosphorylated not only at the Y282 site by MET but also at the Y110 site by the Src family kinase Fyn [20], establishing it as a critical signaling hub within the glioma network. However, since we were unable to successfully generate a specific phospho-antibody against YANK2-Y110, we cannot currently assess the differential expression of these two phosphorylation events in glioma tissues. Nevertheless, functional comparisons based on in vivo tumor growth assays from both studies suggest that the Y110 site may play a more dominant role in YANK2-mediated oncogenic function. Direct side-by-side validation under identical experimental conditions is still warranted to further confirm this notion. Based on our cumulative findings, we propose that Fyn-mediated phosphorylation at Y110 primarily activates YANK2 kinase activity, whereas MET-mediated phosphorylation at Y282 blocks SUMOylation and stabilizes the protein. This multi-kinase regulation suggests that YANK2 may integrate signals from diverse upstream kinases to orchestrate downstream pathways in the tumor microenvironment. These insights provide a novel conceptual framework for understanding the complex signaling landscape of GBM and support the development of multi-targeted combination therapies.

Based on these mechanistic discoveries, we proposed an innovative therapeutic strategy using computer-aided drug design to identify rutin as a specific YANK2-binding compound. While previous studies have shown the ability of rutin to enhance TMZ sensitivity [37–40], our work revealed a novel mechanism of action through direct YANK2 targeting. Structural optimization of rutin could further improve its pharmacokinetic properties [41], potentially enhancing its binding affinity [42] and blood–brain barrier permeability [43]. From a precision medicine perspective, we suggest developing a molecular subtyping system based on YANK2 modification status to better stratify patients for targeted therapies. Monitoring p-Y282 levels and SUMOylation status could serve as valuable biomarkers for patient selection and personalized treatment approaches.

Although this study represents a significant advancement in this field, several critical scientific questions warrant further investigation. First, the intricate crosstalk mechanisms between various post-translational modifications require systematic elucidation. Second, a comprehensive exploration of YANK2’s non-cell-autonomous functions within the tumor microenvironment would provide valuable insights into its broader biological roles. Most importantly, rigorous validation of YANK2-targeting therapeutic strategies in clinically relevant preclinical models is essential before their translational application. Addressing these key questions will facilitate the transformation of YANK2 from a fundamental research discovery to a promising clinical intervention, potentially offering novel therapeutic avenues for patients with glioblastoma.

In conclusion, our study established YANK2 as a novel downstream effector of MET and revealed its intricate post-translational modification network, providing a compelling rationale for the development of new GBM therapies. This mechanism-to-target-to-therapy pipeline provides a valuable framework for precision oncology research. However, critical questions remain regarding (1) the crosstalk between different PTMs, (2) YANK2’s non-cell-autonomous functions in the tumor microenvironment, and (3) its therapeutic potential in preclinical models. Addressing these challenges will facilitate YANK2’s transition from mechanistic discovery to clinically actionable targets, potentially improving outcomes in patients with GBM.

## Materials and methods

### Human glioma specimens

This study included 81 human glioma surgical specimens obtained from the Department of Neurosurgery, Tongji Hospital, Huazhong University of Science and Technology (Wuhan, China). Informed consent was obtained from the patients or their legal guardians prior to sample collection, and the study protocol was approved by the Research Ethics Committee of the same hospital. At least two neuropathologists participated in the histopathological diagnosis of glioma specimens according to World Health Organization (WHO) classification. The pathological grade of the glioma specimens was shown in Supplementary Table 4. All procedures were performed according to the standards of the Helsinki Declaration and approved by the institutional ethics committees of Tongji Medical College of Huazhong University of Science and Technology [44].

### Cell culture

Human glioma cell lines A172, H4, Hs683, U118MG, U373, U251, and U87MG, as well as the murine glioma cell line GL261 were laboratory-owned. The GBM cell line GWH04 [45] was a kind gift from Prof. Dongsheng Guo (Neurosurgery Laboratory, Tongji Hospital). All cell lines were cultured under optimal growth conditions, according to ATCC recommendations. The culture medium was supplemented with 10% fetal bovine serum (FBS) and the cells were incubated at 37 °C in a humidified atmosphere containing 5% CO₂. The culture medium was refreshed every two days. All the cell lines were authenticated via STR profiling and tested for mycoplasma contamination [27].

### Plasmid construction and transfection

The plasmids pCMV-NEDD4L-Flag (QC38815), pCMV-3 × HA-SUMO2 (QC53109), and pCMV-SUMO3-3 × HA (QC51505) were purchased from commercial sources. The pcDNA-His-SUMO1 plasmid was provided by Professor Xiaochuan Wang (Huazhong University of Science and Technology, China). The YANK2-Mcherry plasmid was kindly provided by Professor Liang Wang (Central China Normal University, China). Other constructs, including pcDNA3.1-His-MET, pCMV-His-YANK2-Flag, pHAGE-YANK2-3 × Flag, pET28a-His-YANK2-His, pET23a-His-MET, pLV3-GFP-Luciferase, pMD2.G, psPAX2, pLKO.1, and pCold-TF-p70S6K-His were obtained from laboratory stocks. Mutant constructs (pCMV-His-YANK2-Flag and pHAGE-YANK2-3 × Flag with Y282F, Y282D, K8R, K42R, K148R, K241R, or 5 A mutations) and pCMV-SUMO1-3 × HA were designed and cloned into their respective vectors. The cloning primers used are listed in Supplementary Table 2. To silence genes, shMET (see Supplementary Table 3) was designed and inserted into the pLKO.1 vector to obtain the corresponding plasmids.

For plasmid transfection, 293 T cells were plated at 30–50% confluency. The plasmid DNA was gently mixed with Simple-Fect transfection reagent at a 1:3 (DNA: reagent) ratio in serum-free DMEM, and incubated for 15–20 min at room temperature to allow complex formation. The DNA-reagent mixture was then added dropwise to the cells. After 6 h of incubation, the transfection medium was supplemented with fresh complete medium. The medium was replaced 24 h the post-transfection. Finally, the cells were harvested at 36–48 h for subsequent analysis.

### Establishment of stable cell lines

Lentiviral particles were produced by co-transfecting 293 T cells with the transfer plasmids (pLKO.1, pHAGE, or pLV3) and packaging plasmids (psPAX2 and pMD2.G) at a ratio of 2:1.5:0.5, using Simple-Fect transfection reagent. The viral supernatants were harvested at 48 and 72 h post-transfection, filtered through 0.45 μm membranes, and subsequently used to infect target cells in the presence of 10 μg/mL polybrene for 24 h. Following infection, stable cell lines were established through antibiotic selection with either puromycin (2 μg/mL, YESEN, 60210ES25) for 3–5 days or G418 for 14 days.

### In vitro growth curve analysis by MTT assay

Cell proliferation was evaluated using the MTT colorimetric assay. Briefly, the cells were plated at a density of 1,500 cells/well in 96-well plates and allowed to adhere overnight. The cells were treated with various concentrations of rutin for 48 h. Culture medium was aspirated, and the cells were gently washed twice with phosphate-buffered saline (PBS). Subsequently, 100 μL of MTT solution (0.5 mg/mL in PBS) was added to each well and incubated for 4 h at 37 °C under 5% CO₂. The resulting formazan crystals were solubilized in 200 μL of dimethyl sulfoxide (DMSO) after careful removal of the supernatant. The optical density was measured at 595 nm using a microplate reader. Each experiment was performed in triplicates to ensure reproducibility.

### Colony formation assay

To assess cellular proliferative capacity, cells were seeded at a density of 1,500 cells per well in 6-well plates and cultured under standard conditions (37 °C, 5% CO₂) for 15 days. The culture medium was refreshed every five days to maintain an optimal nutrient supply. Following incubation, the colonies were gently washed with PBS, fixed with 4% paraformaldehyde for 10 min, and stained with 0.1% crystal violet solution for 30 min to visualize the cell clusters. After thorough rinsing with distilled water and air-drying, colony formation was documented by imaging.

### Soft agar colony formation assay

The anchorage-independent growth potential of cells was evaluated using a two-layer soft agar system. Briefly, each well of a 6-well plate was coated with 2 mL of bottom agar (0.5% Bacto-agar, 5% FBS, 0.33% Eagle’s MEM, and 20 μg/mL gentamycin). Subsequently, 1.5 mL of the top agar-cell suspension (0.33% Bacto-agar at 4,000 cells/well) was overlaid. Cultures were maintained at 37 °C with 5% CO₂ for 3–4 weeks, and 1 mL of fresh medium was added weekly to sustain the cell growth. Colony formation, which is indicative of the malignant transformation potential, was assessed using microscopic imaging.

### Western Blotting (WB) and Co-immunoprecipitation (co-IP) assays

For western blot analysis, cells were lysed in radioimmunoprecipitation assay (RIPA) buffer, followed by sonication on ice (10% power output, 3 min). After centrifugation, the supernatant was mixed with the loading buffer and denatured at 95 °C for 15 min. For co-immunoprecipitation experiments, HEK293T or GBM cells were lysed in co-IP buffer (Biosharp) with brief sonication (5% power output). Lysates were incubated with specific antibodies and protein A/G agarose (Santa Cruz Biotechnology) overnight at 4 °C. The immunocomplexes were washed five times with PBS, resuspended in 2 × loading buffer, and boiled at 95 °C for 10 min. Protein samples were separated by SDS sulfate–polyacrylamide PAGE and analyzed by immunoblotting. Detailed antibody information is provided in Supplementary Table 1.

### Bacterial protein expression and purification

Recombinant proteins were expressed in BL21 E. coli cells transformed with the pET28a-His-YANK2(Y282F)-His, pET23a-His-MET or pCold-TF-p70S6K-His plasmids. Bacterial cultures were grown at 37 °C until reaching an OD₆₀₀ of 0.6–0.8, followed by induction with 0.5 mM IPTG at 16 °C overnight. Cells were harvested, washed with PBS, and lysed using three freeze–thaw cycles. After centrifugation, His-tagged proteins were purified from either the supernatant or the solubilized pellet (in 2 M urea) using Ni–NTA agarose (Genscript, L00250). The purified proteins were washed twice with 1 × kinase buffer and stored at −80 °C for subsequent experiments.

### In vitro kinase assay, Immunoprecipitation (IP) kinase assay, and mass spectrometry

Commercially available active MET kinase (sc-2003) and immunoprecipitated active YANK2 were used. For YANK2 preparation, HEK293T cells were transiently transfected with pCMV-His-YANK2-Flag plasmid for 48 h, followed by stimulation with EGF (80 ng/mL) for 30 min. Active YANK2 was purified using Ni–NTA affinity chromatography. Recombinant inactive YANK2, MET, and p70S6K were expressed in bacterial systems and were purified to homogeneity. Kinase reactions were performed in 1 × kinase buffer containing 100 μM ATP at 37 °C for 2 h. Phosphorylation status was assessed by western blotting using specific antibodies against p-Tyr, p-YANK2 Y282, p-Ser/p-Thr, or p-p70S6K, and the phosphorylation sites were further verified by liquid chromatography-mass spectrometry (LC–MS; Thermo Q Exactive Plus, SpecAlly Life Technology).

### Production and purification of p-YANK2 Y282-specific antibody

Custom phosphopeptides (QSVP [pY] LADM) and corresponding non-phosphorylated peptides (QSVPYLADM) were chemically synthesized. The phosphorylated peptide was conjugated to maleimidobenzoyl-N-hydroxysuccinimide ester (MBS)-activated keyhole limpet hemocyanin (KLH) to generate immunogenic complexes. New Zealand White rabbits were immunized subcutaneously with antigens and polyclonal antisera were collected. Peptide-coupled CNBr-activated Sepharose 4B columns were used for affinity purification. Antibodies were sequentially purified by positive selection using a phosphopeptide column, followed by negative depletion using a non-phosphorylated peptide column to remove non-specific binders. Antibody titers were quantified using an indirect ELISA. The affinity-purified antibodies were stabilized in PBS containing 0.02% sodium azide and 50% glycerol and stored at −20 °C for long-term preservation.

### Prediction of potential natural small-molecule inhibitors of YANK2

The YANK2 protein sequence was obtained from the UniProt database. Using AlphaFold, we predicted its three-dimensional structure (YANK2: AF-Q9NY57-F1-v6), followed by binding pocket analysis using Schrödinger SiteMap. The protein was prepared using the Protein Preparation Wizard in Maestro, whereas 3,158 FDA-approved compounds were prepared using LigPrep (Maestro). Molecular docking was conducted using Glide XP in extra precision (XP) mode. The docking results were carefully analyzed through visualization, and representative compounds were selected based on their binding pose quality and volume overlap with the predicted binding pockets.

### Pull-down assay

For the pull-down assay, Rutin or Lactitol (200 mg) was covalently coupled to 0.1 g of CNBr-activated agarose gel 4B beads (GE Healthcare, 17–0430-01), following the manufacturer’s protocol [46]. The successful conjugation of rutin (which exhibits a paleyellow color) to the matrix was confirmed by visual inspection of bead coloration. In the pull-down experiment, 293 T cells were lysed using ice-cold lysis buffer containing 0.01% Nonidet P-40, 0.02 mM PMSF, 150 mM NaCl, 5 mM EDTA, 1 mM dithiothreitol, and 50 mM Tris–HCl (pH 7.5), supplemented with a protease inhibitor cocktail. Cell lysates were incubated overnight at 4 °C with either control or rutin-conjugated beads (containing 2 μg/mL bovine serum albumin as a blocking agent). Following incubation, the beads were subjected to stringent washing with PBS containing increasing concentrations of Tween-20 (0.1%, 0.5%, and 1%). The captured proteins were eluted, separated by SDS-PAGE, and detected by immunoblotting with specific antibodies.

### Cellular thermal shift assay

Based on the principle that drug molecules stabilize target proteins upon binding, we collected U118-YANK2 cells (optimally reaching 5 × 10⁷ cells), washed them three times with PBS containing PMSF, and resuspended the cells in PBS. The cells were then lysed using RIPA buffer. The cell lysate was evenly divided into two aliquots: one was treated with DMSO as a control, and the other was treated with Rutin at a final concentration of 1 μM. The aliquots were incubated at room temperature with rotation for 1 h to allow binding. Subsequently, the samples were evenly distributed into PCR tubes (100 μL per tube). The PCR tubes were placed in a thermal cycler and heated at temperatures ranging from 45 °C to 70 °C, with 5 °C intervals between each group. At each temperature point, the cells were heated for 3 min, then left at room temperature for 3 min, followed by snap-freezing in liquid nitrogen for 3 min; this cycle was repeated twice. After centrifugation at 12,000 rpm for 10 min, the supernatants were collected for sample preparation, and YANK2 expression was detected by western blotting.

### Establishment of intracranial glioma mouse models

Four-week-old male C57BL/6 mice and BALB/c nude mice (purchased from Beijing Vital River Laboratory) were randomly allocated to experimental groups. GL261-Luc cells (2 × 10^5^ cells/10 μL) and GWH04-Luc cells (1 × 10^5^ cells/10 μL) were stereotactically implanted into the right frontal lobe under aseptic conditions. Tumor progression was monitored weekly using in vivo bioluminescence imaging (IVIS) after intraperitoneal injection of D-luciferin (150 mg/kg) under isoflurane anesthesia. Mice were humanely euthanized upon manifestation of neurological deficits in accordance with institutional ethical guidelines. All experimental procedures were approved by the Institutional Animal Care and Use Committee of Tongji Medical College, Huazhong University of Science and Technology, where animals were housed under specific pathogen-free conditions with controlled temperature (22 ± 1 °C) and 12-h light/dark cycles.

### Hematoxylin and eosin staining and Immunohistochemistry (IHC)

Brain tissues were fixed in 4% paraformaldehyde for at least 24 h, followed by standard dehydration through a graded ethanol series, paraffin embedding, and sectioning at 4 μm thickness. After dewaxing and rehydration, the sections were stained with hematoxylin and eosin (HE) for morphological assessment. IHC procedures included:1) endogenous peroxidase inactivation with 3% H₂O₂ for 15 min; 2) antigen retrieval using citrate buffer (pH 6.0) under pressurized heating; 3) non-specific binding blockade with 5% bovine serum albumin (BSA) for 1 h; 4) primary antibody incubation at 4 °C overnight; 5) HRP-conjugated secondary antibody incubation for 1 h at room temperature; 6) diaminobenzidine (DAB) chromogenic development; 7) counterstaining with hematoxylin. All the slides were imaged using an Olympus BX53 microscope equipped with a DP80 camera. Immunohistochemical staining was evaluated independently by three pathologists in a blinded manner. Each section was scored based on two parameters: staining intensity (0, negative; 1, weak; 2, moderate; 3, strong) and the percentage of positive cells (0,0%; 1, 1%−10%; 2, 11%−25%; 3, 26%−50%; 4, 51%−75%; 5, 76%−100%). The final score was calculated as the sum of these two scores, ranging from 0 to 8.

### Statistical analysis

Statistical analyses were performed using unpaired t-tests or one-way ANOVA. Pearson’s correlation coefficient was used to assess the relationships between variables. Data are presented as mean ± SD. Significance was defined as *P < 0.05, **P < 0.01, ***P < 0.001 and ****P < 0.0001. Survival curves were analyzed using the log-rank test, with P < 0.05 considered significant. The data generated in this study are available upon request from the corresponding authors. The authors confirm that the data supporting the findings of this study are available in the article and its supplementary material.

## Supplementary Information

Supplementary Material 1.

## Data Availability

The data that support the findings of this study are available on request from the corresponding author, upon reasonable request.

## Abbreviations

GBM: Glioblastoma
TMZ: Temozolomide
RTKs: Receptor tyrosine kinases
YANK2: Serine/threonine kinase STK32B
MET: Cellular-mesenchymal epithelial transition factor
co-IP: Co-immunoprecipitation
CHX: Cycloheximide
HE: Hematoxylin and eosin
IHC: Hematoxylin and Eosin Staining and Immunohistochemistry
