# Supplementary material for: MET-mediated phosphorylation of YANK2 at Y282 inhibits NEDD4L-dependent SUMOylation and degradation, promoting chemoresistance in glioblastoma
Source: Mol Biomed. 2026 Jun 24;7:95. doi: 10.1186/s43556-026-00496-3 (PMC13291280; doi:10.1186/s43556-026-00496-3)
Supplement: Supplementary file 1 — Supplementary Material 1. [file 43556_2026_496_MOESM1_ESM.pdf]

MET-mediated phosphorylation of YANK2 at Y282 inhibits NEDD4L-dependent SUMOylation and degradation, promoting chemoresistance in Glioblastoma

Yue Shi[1*], Yue Cheng[3,1*], Wensheng Li[1], Annie Zhu[1,6], Juanjuan xiao[2], Wei Wang[4,1], Liu Tang[1], Shuang Zhao[2], Mee-Hyun Lee[5], Olesya S. Malyarenko[2], Qiuhong Duan[1, 2#]

1. Department of Biochemistry and Molecular Biology, School of Basic Medicine, Tongji Medical College, Huazhong University of Science and Technology, Wuhan, Hubei, 430030, China.

2. Translational Medicine Center, Huaihe Hospital of Henan University, Henan University, Kaifeng, Henan, China.

3. Department of Clinical Laboratory, Zhengzhou Eighth People's Hospital, Zhengzhou, Henan, China.

4. Wuhan Fourth Hospita, 430030, Wuhan China.

5. College of Korean Medicine, Dongshin University, Naju, 58245, South Korea

Electronic

6.Phillips Exeter Academy, PEA 2211, 20 Main Street, Exeter, NH03833, USA

Corresponding author: Qiuhong Duan, duanqhwz@henu.edu.cn, ORCID: 0009-0005-7763-9691, 0086-18971564406, 430030

* These authors contributed equally.

Yue Shi, 1281842566@qq.com

Yue Cheng, 969127768@qq.com

Wensheng Li, 2628220830@qq.com

Annie Zhu, azzhu@exeter.edu

Juanjuan xiao, xjjuan2006@henu.edu.cn

Wei Wang, 489622753@qq.com

Liu Tang, 1490753262@qq.com

Shuang Zhao, zaosuangh@henu.edu.cn

Mee-Hyun Lee, mhyun_lee@hanmail.net

Olesya S. Malyarenko, malyarenko.os@gmail.com

Conflicts of Interest: The authors declare no potential conflicts of interest

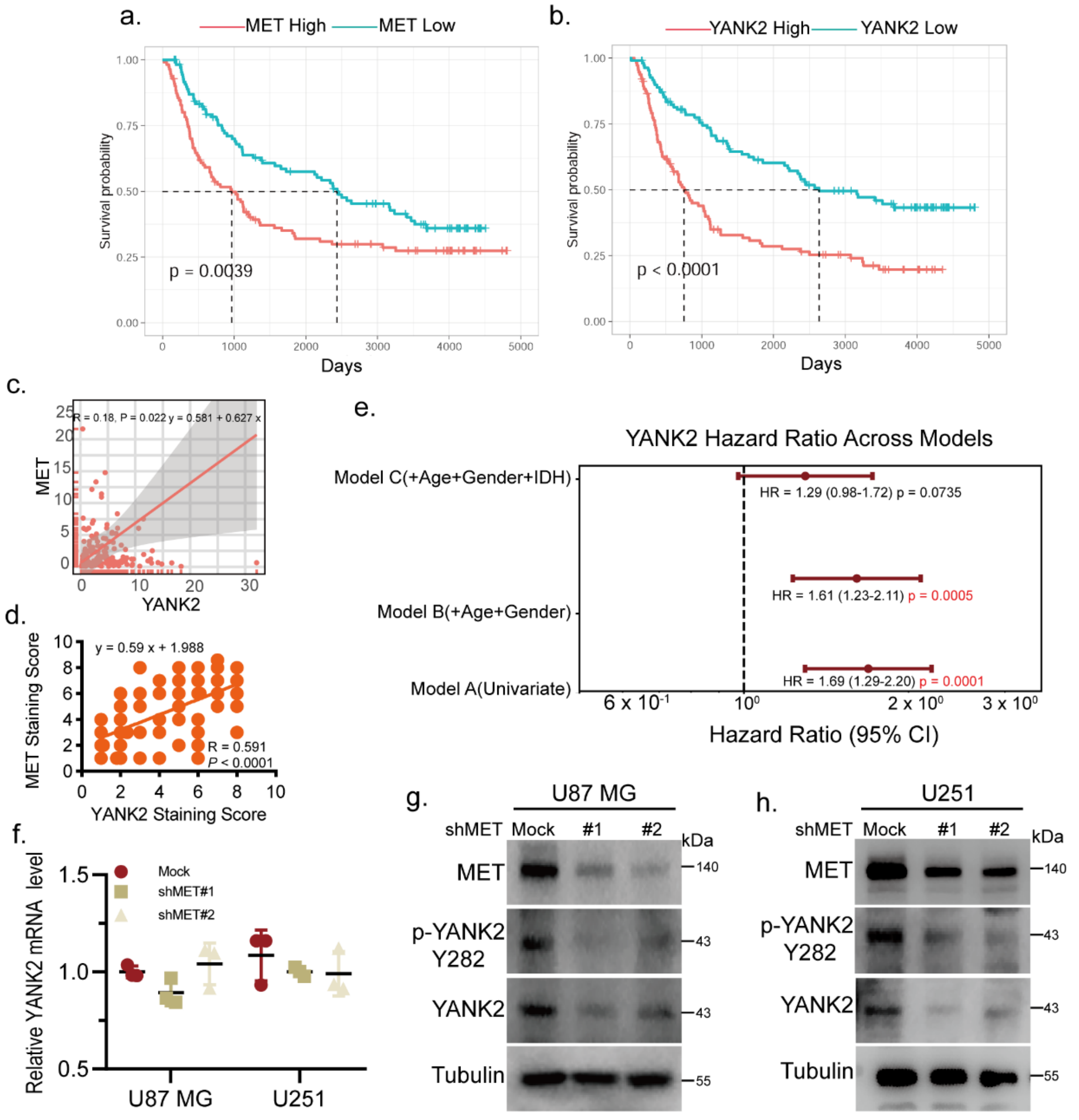

Figure S1. High expression of MET and YANK2 in glioma patients is associated with poor prognosis.

(a) Kaplan–Meier survival curves of GBM patients stratified by YANK2 mRNA levels in the CGGA dataset.

(b) Kaplan–Meier survival curves of GBM patients stratified by MET mRNA levels in the CGGA dataset.

(c) Correlation analysis of YANK2 and MET expression in human GBM tissues based on the CGGA dataset.

(d) Correlation analysis of YANK2 and MET expression in human GBM tissues based on the IHC score in 81 patients.

(e) Multivariate Cox regression analysis of YANK2 expression and overall survival in GBM patients adjusted for age and IDH mutation status. Hazard ratios (HRs) and 95% confidence intervals (CIs) were calculated using the CGGC database. Age and IDH mutation status were included as covariates to exclude their confounding effects. Statistical significance was defined as $P < 0.05$.

(g-h) Western blot analysis of YANK2 and p-YANK2 protein levels in U87 MG and U251 shMET glioma cell lines.

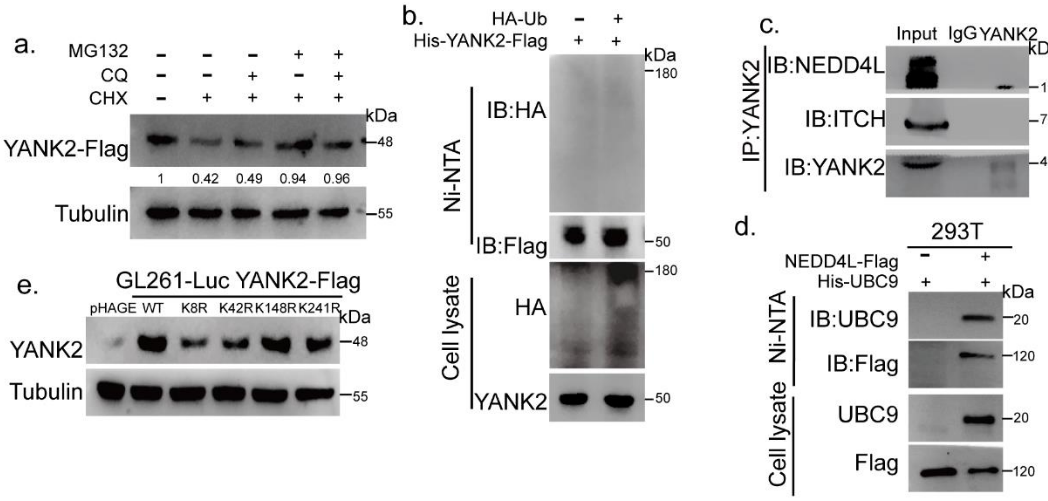

Figure S2 The degradation mechanism of YANK2 and proteins interacting with NEDD4L.

(a) WB analysis of YANK2 protein levels in cells treated with MG132 (10 μM, 4 h), CQ (10 μM, 4 h), or CHX.

(b) WB analysis of YANK2 ubiquitination in HEK293T cells co-transfected with His-YANK2-Flag and HA-Ub plasmids.

(c) Endogenous co-IP of YANK2 in whole lysates from U87MG cells using anti-YANK2 or IgG, followed by WB.

(d) Co-immunoprecipitation (co-IP) of UBC9-His and NEDD4L-Flag in HEK293T cells using Ni-NTA or anti-Flag beads, followed by WB analysis.

(e) Western blot analysis of YANK2 levels in GL261-Luc glioma cell lines with YANK2 overexpression.

a.

Rutin hydrate

b.

Lactitol

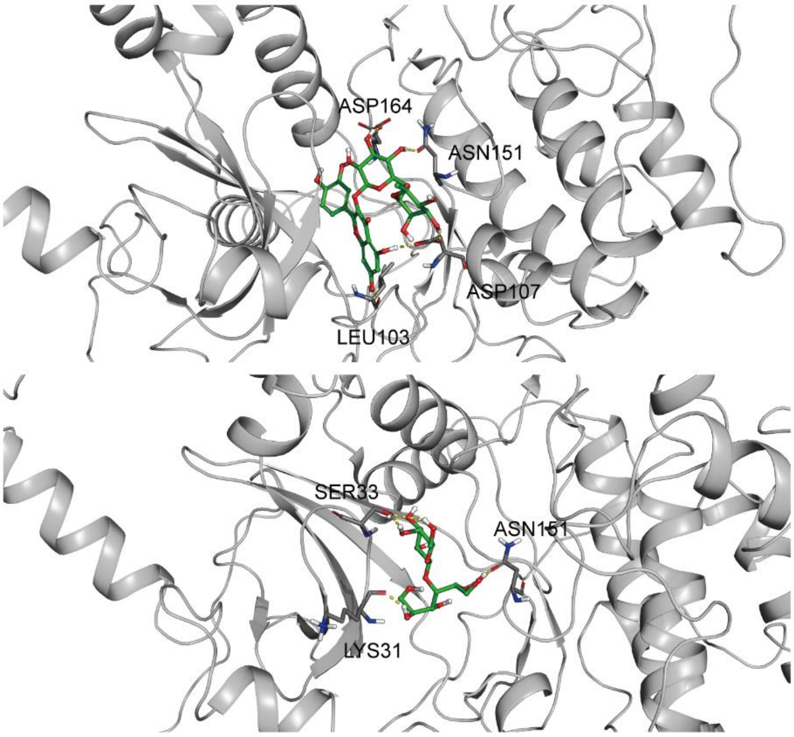

Figure S3: Small molecule compounds binding to YANK2

(a-b) 3D interaction diagrams of the top two compounds (colored) docked with YANK2 (gray), including Rutin Hydrate (a) and Lactitol (b).

The three-dimensional structure of YANK2 was obtained by AlphaFold prediction under the entry ID AF-Q9NY57-F1-v6.

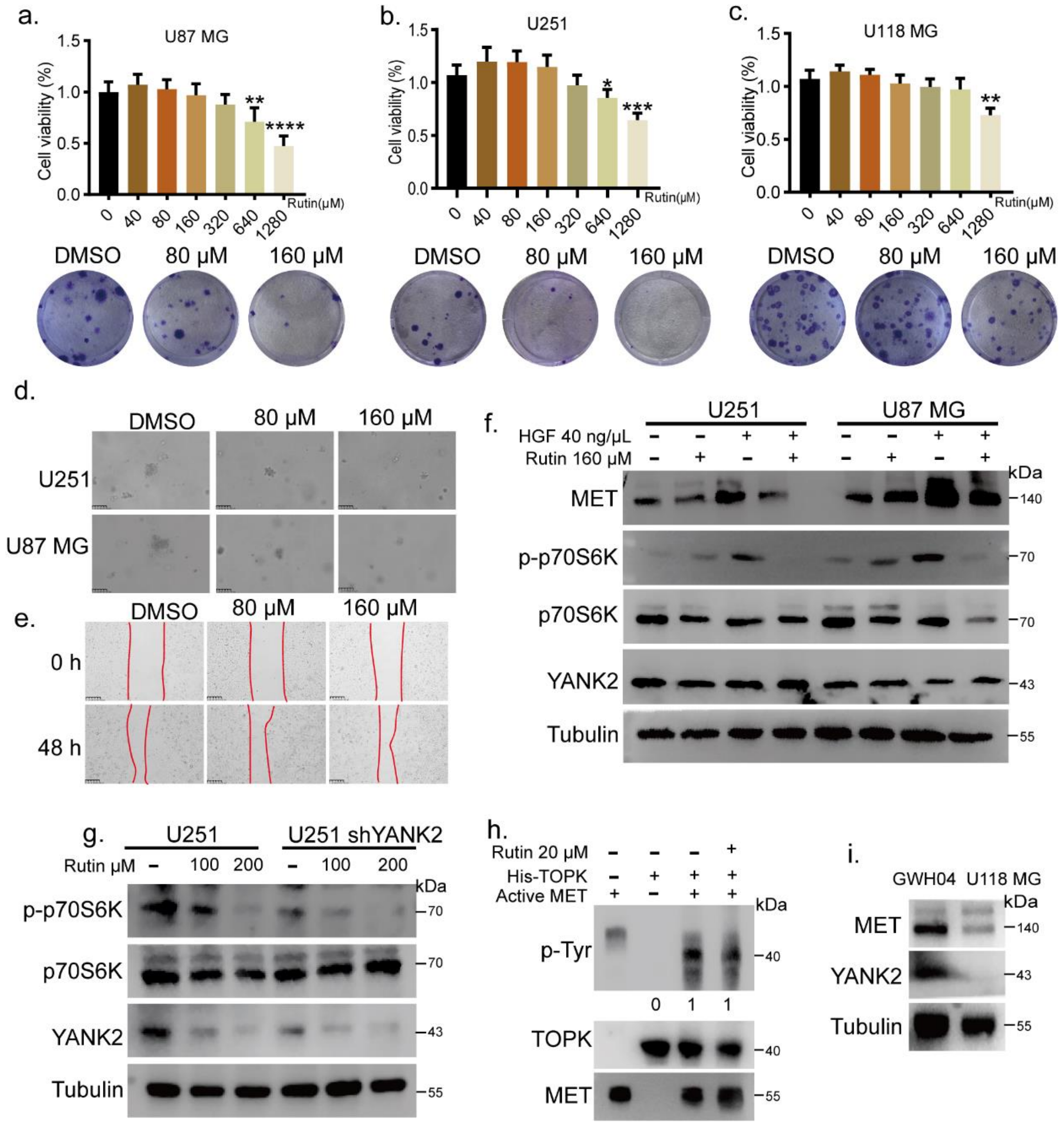

Figure S4. Rutin suppresses glioma cell proliferation and YANK2-p70S6K.

(a–c) MTT and colony formation assays demonstrating the antiproliferative effects of Rutin on U251, U87MG, and U118MG cells.

(d) Soft agar assays confirming the effects of rutin on 3D cultures of U251 and U87MG cells, Scale bars: 100 µm.

(e) Effect of rutin on GWH04 glioma cell migration detected by wound healing assay. Images were taken at 0 h and 48 h post-treatment, Scale bars: 100 µm.

(f) WB analysis of p-p70S6K levels after Rutin or HGF treatment in U251 and U87MG cells.

(g) WB analysis of p-p70S6K levels after Rutin in U251-Mock and U251-shYANK2 cells.

(h) In vitro kinase assays showing that rutin affects MET-mediated phosphorylation of TOPK.

(i) WB analysis of MET, YANK2 and Tubulin in GWH04 and U118MG cells.

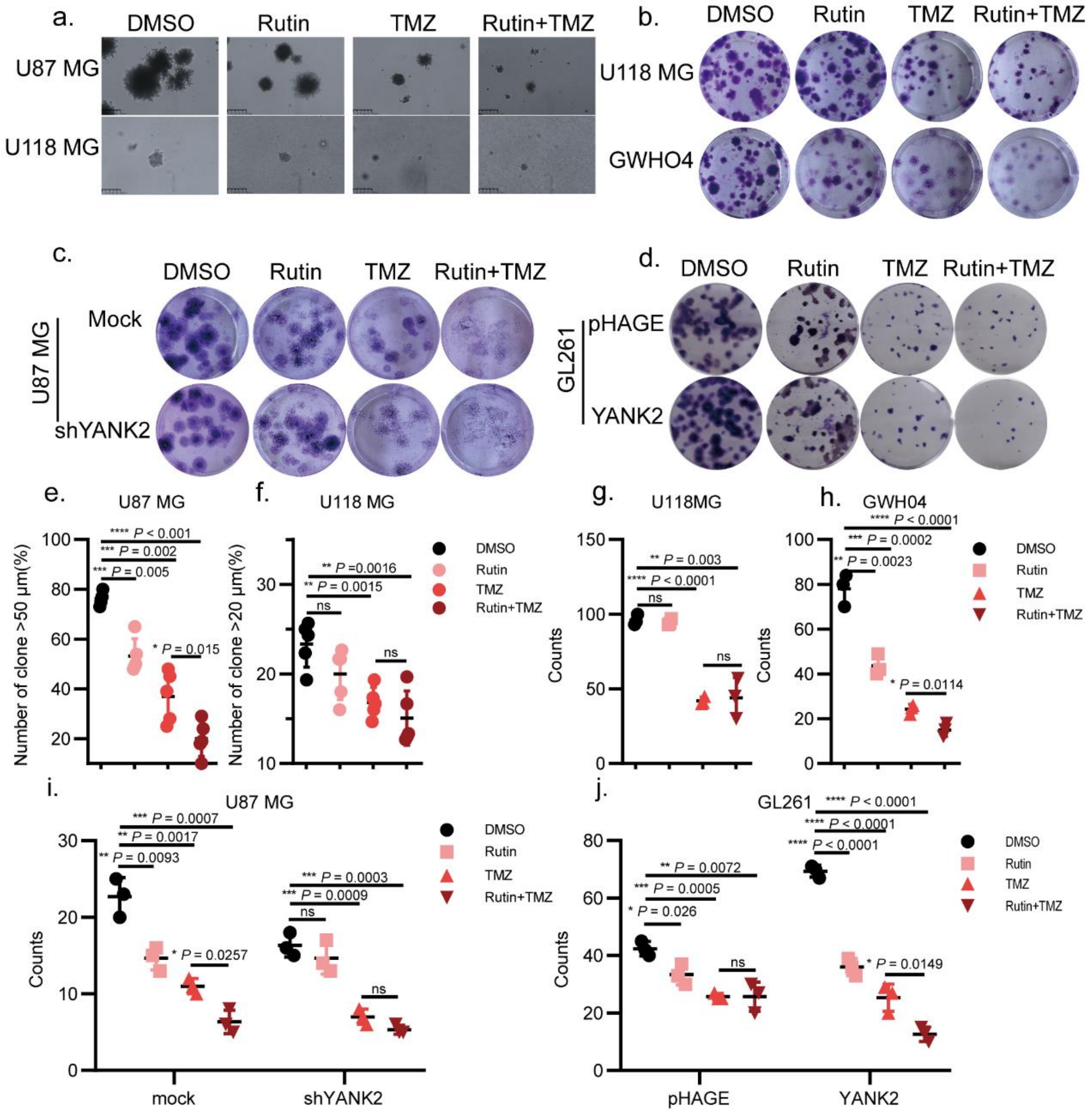

Figure S5. Combination treatment with Rutin and TMZ synergistically inhibits proliferation of YANK2-high glioma cells in vitro.

(a) Soft agar assay showing the effects of rutin (160 μM), TMZ (300 μM), and their combination on U87MG and U118MG cells and quantification of colonies, Scale bar: 100 μm.

(b) Colony formation assay showing the effects of rutin (160 μM), TMZ (300 μM), and their combination on GWH04 and U118MG cells and quantification of colonies.

(c-d) Colony formation assay the effects of rutin (160 μM), TMZ (300 μM), and their combination on GL261-YANK2 cells and U87MG-shYANK2.

(e-f) Quantitative analysis of YANK2-high glioma cell proliferation following treatment with rutin, TMZ, or their combination in a.

(g-h) Quantitative analysis of YANK2-high glioma cell proliferation following treatment with rutin, TMZ, or their combination in b.

(i) Quantitative analysis of YANK2-high glioma cell proliferation following treatment with rutin, TMZ, or their combination in c.

(j) Quantitative analysis of YANK2-high glioma cell proliferation following treatment with rutin, TMZ, or their combination in d.

Table S1. A list of antibodies

Antibodies	Source	Cat. No.	Dilution
Anti-HA-tag(R)	Biodragen	B2229	1：2000
Anti-His-tag(R)	Cell Signaling Technology	#2365	1：2000
Anti-Flag-tag(M)	Sigma-Aldrich	F1804	1：4000
Anti-Flag-tag(R)	Sigma-Aldrich	F7425	1：4000
Anti-YANK2(R)	Abcam	Ab154657	1：3000
Anti-YANK2(R)	Proteintech	15438-1-AP	1：2000
Anti-MET(M)	Cell Signaling Technology	#8198	1：2000
Anti-MET(R)	Invitrogen	37-0100	1：2000
Anti-p70S6K(R)	Cell Signaling Technology	#9202	1：2000
Anti-p70S6K(M)	Proteintech	66638-1-Ig	1：1000
Anti-p-p70S6K(R)	Cell Signaling Technology	#9205	1：3000
Anti-Tubuin(R)	Proteintech	10068-1-AP	1：1000
Anti-Tubuin(M)	Proteintech	66031-1-Ig	1：1000
Anti-NEDD4L(M)	Proteintech	67276-1-Ig	1：1000
Anti-SUMO1(M)	Cell Signaling Technology	#4930	1：2000
Anti-Ub(M)	Abclonal	A19686	1：1000
Anti-p-Tyrosine(R)	Cell Signaling Technology	#8954	1：2000
Anti-p-Serine/Threonine(R)	Abcam	ab300625	1：2000
Anti-p-Threonine(R)	Millipore	AB1607	1：2000

Table S2. A list of primers for mutant plasmids construction

Primer	Sequence
WT-R(Xhol)	GGATCCAAGCTTCGCCCGGGCCAGATCTTCC
WT-F(Hind III)	CCCAAGCTTATGGGCGGGAACCACTCCCAC
WT-F(MiuI)	CGACGCGTATGGGCGGGAACCACTCCCAC
Y282F-F	GACATACAGAGCGTGCCCTTCTTGGCCGACATGAACTGG
Y282F-R	CCAGTTCATGTCGGCCAAGAAGGGCACGCTCTGTATGTC
Y282D-F	GACATACAGAGCGTGCCCGACTTGGCCGACATGAACTGG
Y282D-R	CCAGTTCATGTCGGCCAAGTCGGGCACGCTCTGTATGTC
K8R-F	GGCGGGAACCACTCCCACAGGCCCCCCGTGTTTGACGAG
K8R-R	CTCGTCAAACACGGGGGGCCTGTGGGA

	GTGGTTCCCGCC
K42R-F	GAAAGGTATGCATCGTGCAGAGGCGAGACACTAAGAAAATGTATGC
K42R-R	GCATACATTTTCTTAGTGTCTCGCCTCTGCACGATGCATACCTTTC
K148R-F	CACATCATCCACAGAGACATCAGGCCAGACAATATCCTGCTGG
K148R-R	CCAGCAGGATATTGTCTGGCCTGATGTCTCTGTGGATGATGTG
K241R-F	CGATGAAATCCTCAACATGTTCAGGGTGGAGCGTGTCCACTACTC
K241R-R	GAGTAGTGGACACGCTCCACCCTGAACATGTTGAGGATTTCATCG
E71A-F	GTTCGGAATGTTTTCCGGGCGCTGCAGATCATGCAAGGGC
E71A-R	GCCCTTGCATGATCTGCAGCGCCCGGAAAACATTCCGAAC
L103S-F	GTTCATGGTGGTGGACCTGAGCCTGGGAGGCGCCCTGCGCTACCATCTGCAG
L103-R	CTGCAGATGGTAGCGCAGGGCGCCTCCCAGGCTCAGGTCCACCACCATGAAC
D150A-F	CCACAGAGACATCAAGCCAGCCAATATCCTGCTGGATGAACAC
D150A-R	GTGTTCATCCAGCAGGATATTGGCTGGCTTGATGTCTCTGTGG
D164A-F	CACGGACATGTTCACATTACAGCCTTCAACATAGCGACGGTAGTG
D164A -R	CACTACCGTCGCTATGTTGAAGGCTGTAATGTGAACATGTCCGTG
SUMO1-F	CCCAAGCTTATGTCTGACCAGGAGGCAAAACC
SUMO1-R	CCGCTCGAGCTAAACTGTTGAATGACCCCCCG

Table S3. A list of primers for target sequences for shRNA

shRNA	Order	Target Sequence
MET	#1	GAAGTCCTCTTAACATCTATA
	#2	GCTGTGAGAATATACACTTAC
YANK2	#1	CAGAAGCGAGACACTAAGAAA
	#2	GCAGCAGAATGTGCATTTCAC

Table S4. A list of Pathological grade

Num	Row	Column	Gender	Age	Pathological diagnosis: (0 - Meningioma/benign lesion; 1- Pilocytic astrocytoma glial hyperplasia; 2-4 - Glioma)
1	1	1	Male	63	2
2	1	2	Male	47	4
3	1	3	Male	33	3
4	1	4	Male	44	4
5	1	5	Female	73	4
6	1	6	Male	55	4
7	1	7	Male	63	4
8	1	8	Male	58	4
9	1	9	Female	47	2
10	1	10	Female	44	2
11	1	11	Male	65	4
12	1	12	Male	68	4
13	2	1	Male	35	4
14	2	2	Female	42	2
15	2	3	Male	30	4
16	2	4	Female	35	2
17	2	5	Female	53	2
18	2	6	Male	48	4
19	2	7	Male	47	2
20	2	8	Female	44	2
21	2	9	Male	46	2
22	2	10	Female	43	3
23	2	11	Male	60	4
24	2	12	Male	65	4
25	3	1	Female	56	3
26	3	2	Female	50	2.3
27	3	3	Female	50	4
28	3	4	Male	50	4
29	3	5	Female	42	4
30	3	6	Male	45	3
31	3	7	Male	44	4
32	3	8	Male	46	4
33	3	9	Female	51	4
34	3	10	Male	65	4
35	3	11	Male	62	4
36	3	12	Female	36	2
37	4	1	Female	35	4
38	4	2	Male	30	4
39	4	3	Male	65	4

40	4	4	Male	11y5m	4
41	4	5	Female	16	1
42	4	6	Female	26	4
43	4	7	Male	43	4
44	4	8	Male	61	4
45	4	9	Female	38	2
46	4	10	Female	38	4
47	4	11	Female	54	4
48	4	12	Male	46	4
49	5	1	Female	48	3
50	5	2	Male	31	2
51	5	3	Female	47	3
52	5	4	Male	55	4
53	5	5	Female	37	2.3
54	5	6	Male	53	4
55	5	7	Male	47	2
56	5	8	Male	31	2
57	5	9	Male	49	4
58	5	10	Female	50	4
59	5	11	Female	9	3
60	5	12	Male	46	4
61	6	1	Female	62	4
62	6	2	Male	1y6m	3.4
63	6	3	Female	32	2.3
64	6	4	Female	55	4
65	6	5	Female	55	3
66	6	6	Male	34	2
67	6	7	Female	34	2
68	6	8	Male	69	4
69	6	9	Female	57	4
70	6	10	Male	17	3
71	6	11	Male	45	2
72	6	12	Female	59	4
73	7	1	Female	55	3.4
74	7	2	Female	59	2.3
75	7	3	Male	48	2
76	7	4	Male	52	2
77	7	5	Male	40	2
78	7	6	Male	56	4
79	7	7	Female	61	2
80	7	8	Male	1y8m	2
81	7	9	Male	16	3
